# Effect of Air Gap on Electrical Tree in Epoxy Resin Under High Frequency Bipolar Square-Wave Voltage

**DOI:** 10.3390/ma13245722

**Published:** 2020-12-15

**Authors:** Shihang Wang, Chuang Zhang, Hang Fu, Jiao Xiang, Jianying Li, Shengtao Li, Benhong Ouyang, Jianben Liu

**Affiliations:** 1 State Key Laboratory of Power Grid Environmental Protection, China Electric Power Research Institute, Wuhan 430074, China; ouyangbenhong@epri.sgcc.com.cn (B.O.); liujianben@epri.sgcc.com.cn (J.L.); 2 State Key Laboratory of Electrical Insulation and Power Equipment, Xi’an Jiaotong University, Xi’an 710049, China; zhangchuang@stu.xjtu.edu.cn (C.Z.); fh291009595@stu.xjtu.edu.cn (H.F.); lijy@mail.xjtu.edu.cn (J.L.); sli@mail.xjtu.edu.cn (S.L.); 3 Power China Hubei Electric Engineering Co., Ltd., Wuhan 430000, China; xiangtian886633@stu.xjtu.edu.cn

**Keywords:** epoxy resin, electrical tree, air gap, high voltage, high frequency

## Abstract

Insulation fails quickly under high-frequency AC high voltage, especially bipolar square-wave voltage with a high d*V*/d*t*. It is of great significance to study the failure mechanism of epoxy casting insulation under such kind of voltage. In this paper, pin-plane epoxy casting insulation samples with air gaps were prepared, and the relation between the electrical trees under the high frequency bipolar square-wave voltage and the air gap conditions and voltage frequencies (1~20 kHz) were studied. Results indicated that, with the presence of air gaps, the electrical trees were bush-type and had a relatively slow growth rate, which was different from the fast-growing branch-type trees in the samples without air gap. The electrical tree characteristics related with the size of air gap and voltage frequency were also studied. The electrical tree grew faster under higher voltage frequency or with a smaller air gap. Results proved that discharge introduced a lot of defects for the surface layer of the epoxy resin samples and hence induced the possibility of multi-directional expansion of electrical trees. In addition, the resulting heat accumulation and unique charge transport synergistically affected the electrical tree characteristics under the high frequency bipolar square-wave voltage.

## 1. Introduction

The solid-state transformer (SST) has been regarded as one of the 10 most emerging technologies by Massachusetts Institute of Technology (MIT) Technology Review in 2010 and has received widespread research in recent years [1,2,3,4]. Insulation problems in solid-state transformers are getting more and more attention, mainly because the insulation dielectrics are prone to failure and have a relatively short service life. Epoxy casting insulation is the primary insulation of the winding structure in solid-state transformers, and it is subjected to the high frequency bipolar square-wave voltage (1 kHz to 100 kHz) and high temperature (about 100 °C) [3,4]. Therefore, it is very important to clarify the aging and failure mechanism of epoxy casting insulation under high-frequency bipolar square-wave voltage.

Electrical tree is an important phenomenon for studying the long-term breakdown of solid insulation. The electrical tree in epoxy resin under 50 Hz AC voltage and DC voltage has been widely studied, but there is little research on the electrical treeing under bipolar square-wave voltage with high frequency [5,6,7,8,9]. Generally speaking, the needle tip structure in the research of electrical tree can simulate the conductor tip defects in the insulation of electrical equipment, such as the protuberance of semi-conducting shielded layer in power cable [10]. However, in epoxy casting insulation, the air gap defects are more likely to exist than the metal tip defects. Therefore, the effect of air gap should be considered to better study the electrical treeing failure of epoxy casting insulation under high-frequency voltage.

The morphology of electrical treeing in epoxy casting insulation is quite different in different literatures [11,12,13,14], including twig-type tree, branch-type tree, pine-like tree, bush-type tree and hybrid tree. This is mainly influenced by the material characteristics, the voltage waveform and the test conditions. The existence of the air gap further increases the complexity of electrical treeing in epoxy matrix [15,16,17,18]. Some reports showed that the air gaps reduced the tree initiation voltage by about 50% under DC voltage [18], initiated trees quicker under 50 Hz AC voltage, and influenced the subsequent tree growth [15]. It has been proved that the discharge phenomenon is more likely to occur when the frequency of the AC voltage increases, accompanied with the temperature rise [19]. The existence of the air gap allows enough space for discharge, and thus the electrical tree initiated from air gap defect is more complicated [20].

Therefore, in order to be more suitable for service conditions, this paper studied the electrical treeing in epoxy casting insulation under the action of repetitive frequency bipolar square-wave voltage with the cone-shaped air gaps, which has not been reported yet.

## 2. Materials and Methods

The studied epoxy resin is a bisphenol-A resin (E51). The methyl tetrahydrophthalic anhydride hardener (MTHPA) was used as a curing agent at 80 phr. Tris (dimethylaminomethyl) phenol (DMP-30) was used as an accelerator at 1 phr. The above three raw materials are uniformly mixed by mechanical stirring. The mixture was then stirred and placed in a vacuum chamber for 20 min, to avoid bubbles in the epoxy samples after pouring.

**Figure 1:**
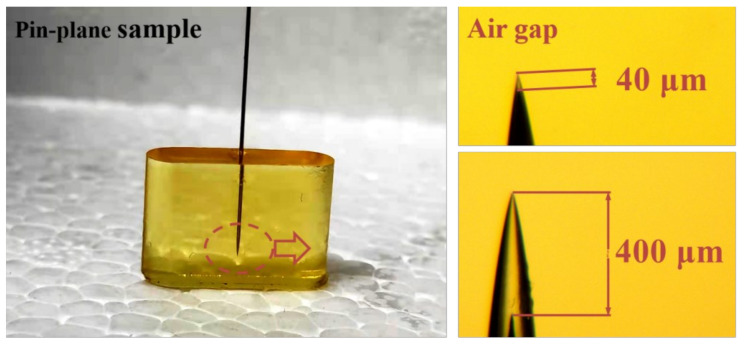
Picture of epoxy resin sample with needle and air gap.

The size of the mold for casting epoxy samples was 20 × 15 × 5 mm. The steel needles with same tip radius (2 μm) were placed in each mold in advance, and the needle tip was 2 mm away from the bottom of the mold. The epoxy resin was cured at the mold at 80 °C for 2 h, 105 °C for 2 h and 120 °C for 4 h. In order to prevent the sample from cracking, the cooling rate of the sample should not be too fast after curing. Before the electrical tree experiments, the needles in the samples were drawn out a fixed distance, 40 or 400 μm, respectively. As a result, the cone-shaped air gaps were formed around the needle tips. Then the relative position of the needle and the epoxy sample was fixed. The pin-plane epoxy resin sample with needle and air gap is shown in the Figure 1.

**Figure 2:**
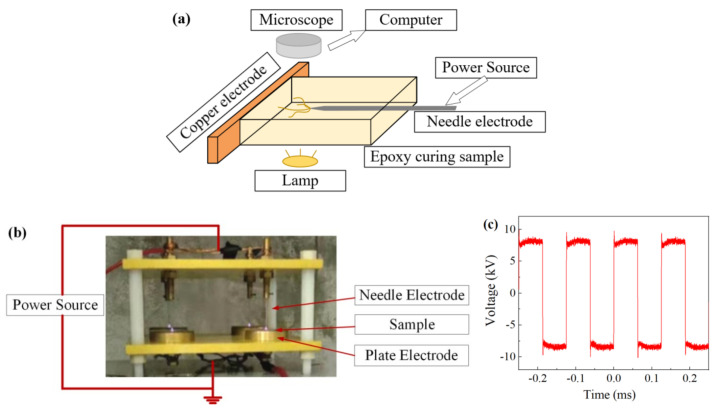
Schematic diagram of test platform and conditions, (**a**) the observation platform of electrical tree, (**b**) the corona test platform, (**c**) the voltage waveform.

An observation platform for electrical tree was built, as shown in Figure 2a. The high-voltage power source can continuously output bipolar square-wave high voltage, and the frequency can be continuously adjusted between 1 and 25 kHz. The needle was connected to the high voltage and the other side was connected to the grounded copper electrode. A microscope with a camera was used to observe the electrical treeing and a computer was used to record the images. The electrical tree test for each kind of sample and each voltage waveform was done three times to ensure the repeatability of the results.

A corona test platform was built to test the damage characteristics of the corona discharge on the epoxy samples under high frequency bipolar square-wave voltage, as shown in Figure 2b. These two platforms have the same pin-plane electrode system. Epoxy resin sheet samples with thickness of 0.4 mm were used to study the discharge damage. The corona test was done three times to ensure the accuracy of the results. The BX51-P polarizing microscope was applied to observe the surface morphology of the samples after corona aging. The PGI3D aspheric surface measuring instrument was used to measure the surface contour curve of the samples.

The voltage amplitude has a great influence on the growth of electrical tree, and the voltage in the electrical tree experiments was fixed at ±8 kV. The voltage waveform (take 8 kHz/8 kV as an example) is shown in Figure 2c.

## 3. Results

### 3.1. Electrical Treeing in Epoxy Casting Insulation Samples without Air Gap

**Figure 3:**
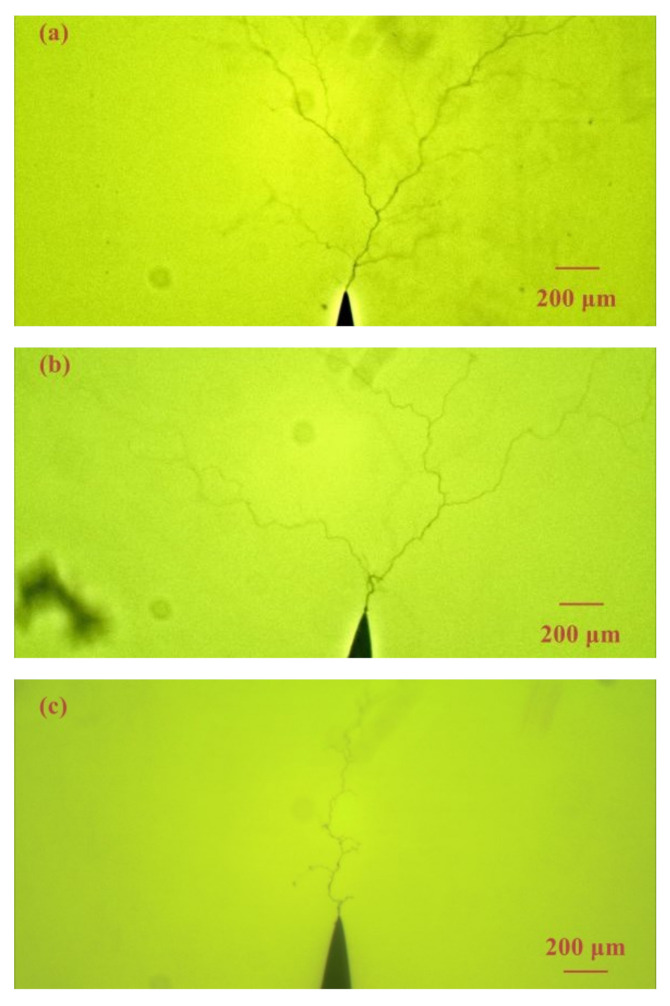
Electrical tree morphology in epoxy casting insulation samples without air gap under 8 kV bipolar square-wave voltage with frequency of (**a**) 1 kHz, (**b**) 8 kHz, (**c**) 20 kHz.

In order to better analyze the electrical tree characteristics in epoxy casting insulation samples with air gaps, the electrical treeing initiated from needle tip was first analyzed. Figure 3 shows the electrical tree morphology in epoxy resin sample without air gap under ±8 kV bipolar square-wave voltage with frequency of 1 kHz, 8 kHz and 20 kHz after 2 min. The obvious difference in electrical treeing under different frequencies is the branch number. The electrical tree under 1 kHz is a typical branch-like tree, and when the frequency increased to 8 kHz, the branches of the electrical tree are significantly reduced. Until under 20 kHz, the electrical tree has only one main channel and several inconspicuous small branches. The number of branches determines the width of electrical tree. As shown in Figure 3c, the width of the electrical tree under 20 kHz is less than 200 μm, which is much smaller than the other two cases. From the perspective of growth rate, the electrical trees penetrated to the ground electrodes in about 200 s at these three frequencies. When the electrical trees contacted the ground electrode, the samples did not breakdown directly but would breakdown after a period of time. In order to protect the power supply and avoid multiple short circuits, the voltage was removed immediately, and the test was ended when the electrical tree reached the ground electrode.

### 3.2. Electrical Treeing in Epoxy Casting Insulation Samples with Air Gaps

**Figure 4:**
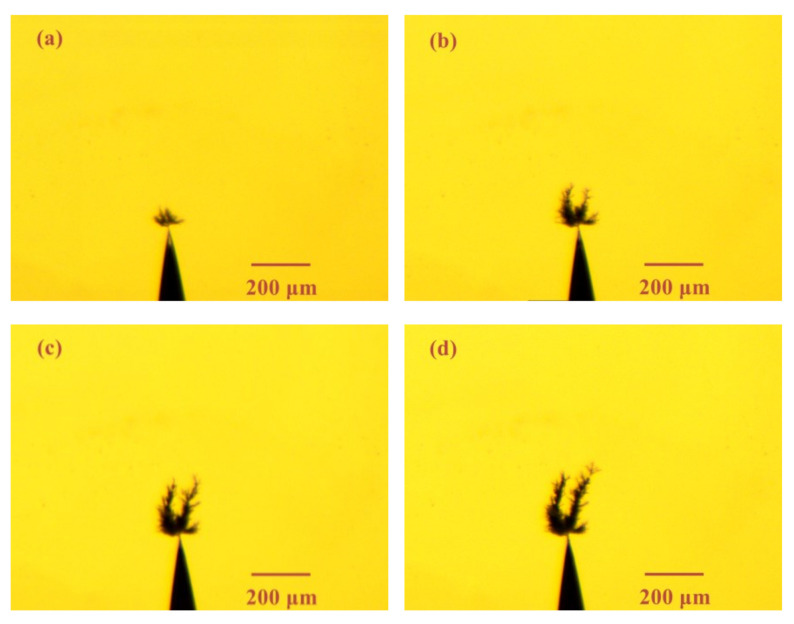
Electrical tree morphology in epoxy casting insulation sample with small air gap under 1 kHz/8 kV bipolar square-wave voltage at time of (**a**) 1 min, (**b**) 5 min, (**c**) 10 min, (**d**) 15 min.

Figure 4 shows the electrical tree morphology in epoxy resin sample with a small air gap (length: 40 μm) under 1 kHz/8 kV bipolar square-wave voltage. The tree growth was recorded within 15 min. In the presence of an air gap, the electrical tree characteristics has a very large change. On the one hand, the growth rate has slowed down significantly. On the other hand, the morphology of the electrical tree becomes bush-like.

During the preparation of electrical tree samples, due to the different thermal expansion coefficients of the epoxy casting insulation and the needles, an air gap may be generated during the cooling process of the sample. For example, it has been reported the existence of an air gap with size of about 26 μm in the electrical tree sample [20]. However, in our paper, the electrical tree morphology of the sample without air gap and the sample with small air gap is very different, which can indirectly prove that the needle and the epoxy resin matrix are in good contact in the samples without air gaps.

**Figure 5:**
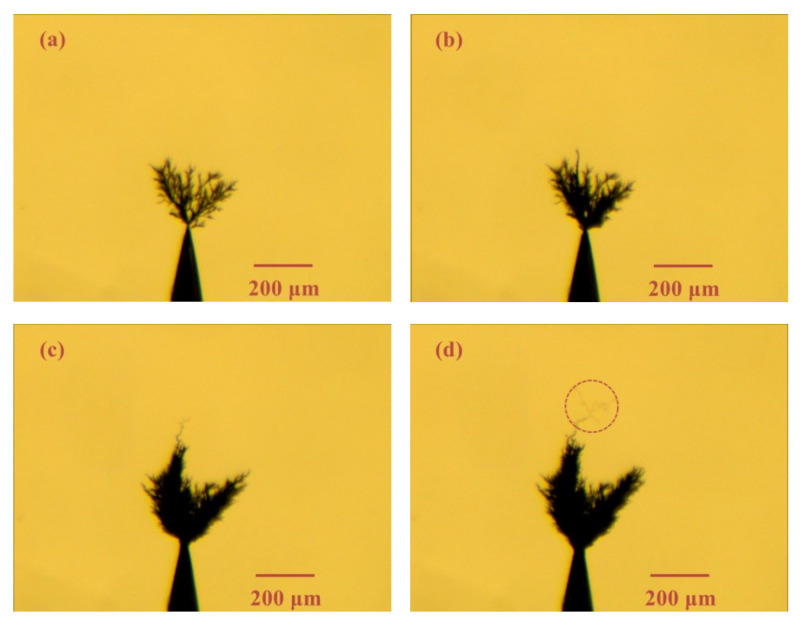
Electrical tree morphology in epoxy casting insulation sample with small air gap under 8 kHz/8 kV bipolar square-wave voltage at time of (**a**) 1 min, (**b**) 5 min, (**c**) 10 min, (**d**) 15 min.

**Figure 6:**
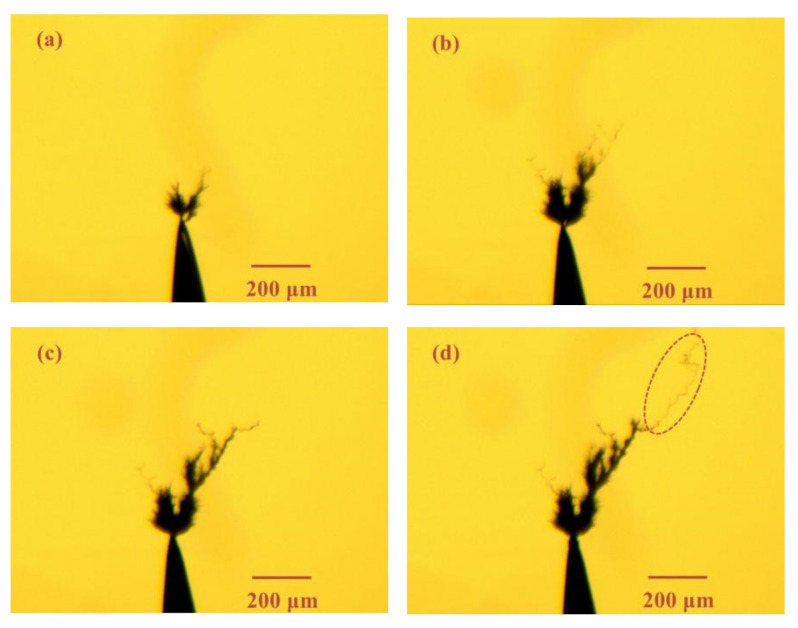
Electrical tree morphology in epoxy casting insulation sample with small air gap under 20 kHz/8 kV bipolar square-wave voltage at time of (**a**) 1 min, (**b**) 5 min, (**c**) 10 min, (**d**) 15 min.

Figure 5 and Figure 6 show the electrical tree morphology in epoxy casting insulation sample with small air gap under 8 and 20 kHz bipolar square-wave voltage, respectively. After the frequency increases, there are two main changes in the electrical tree morphology. One is that the growth rate has increased. In addition, it is found that the growth of the electrical tree has a stagnant period. After this stagnation period, the further extension of the electrical tree is in the form of new branches, as shown by the red dotted line in Figure 5 and Figure 6.

**Figure 7:**
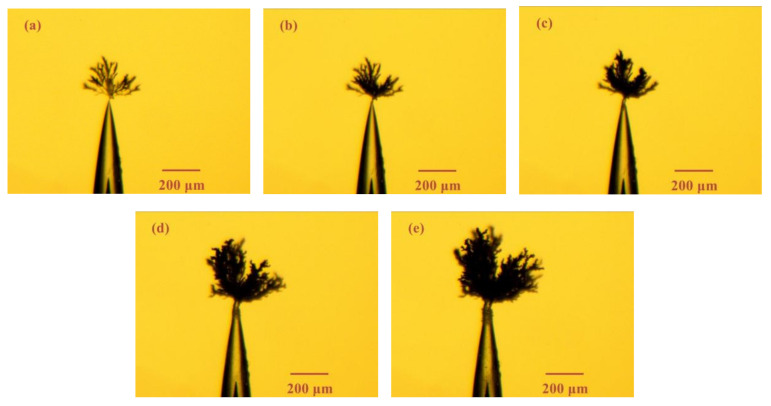
Electrical tree morphology in epoxy casting insulation sample with big air gap under 1 kHz/8 kV bipolar square-wave voltage at time of (**a**) 5 min, (**b**) 20 min, (**c**) 40 min, (**d**) 120 min, (**e**) 240 min.

Figure 7 shows the electrical tree morphology with large air gap (length: 400 μm) under 1 kHz bipolar square-wave voltage. When there is a larger air gap, electrical trees have new characteristics. Electrical tree grows more slowly and tend to carbonize and erode the surrounding dielectrics, rather than tend to expand. Electrical trees are thicker, and the hairy electrical trees are rare. It is believed that there is corona discharge in the air gap. The samples with large air gap have enough space to observe the corrosion effect of the discharge on the epoxy resin surface in the gap. After applying voltage for 120 min, black spots can be seen in the air gap, which is considered to be caused by discharge erosion of the epoxy resin surface. This phenomenon becomes more obvious at 240 min, as shown in Figure 7e. Another phenomenon is that a thicker channel appears at the tip of the air gap where the trees are triggered.

**Figure 8:**
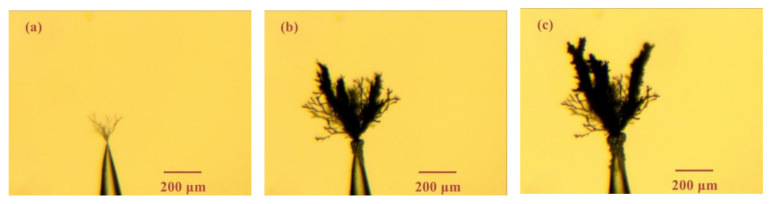
Electrical tree morphology in epoxy resin sample with big air gap under 8 kHz/8 kV bipolar square-wave voltage at time of (**a**) 5 min, (**b**) 20 min, (**c**) 40 min.

**Figure 9:**
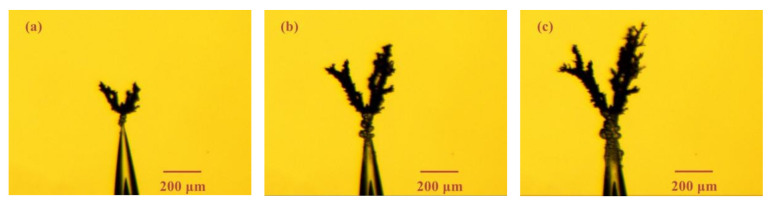
Electrical tree morphology in epoxy casting insulation sample with big air gap under 20 kHz/8 kV bipolar square-wave voltage at time of (**a**) 5 min, (**b**) 20 min, (**c**) 40 min.

Figure 8 and Figure 9 show the electrical tree morphology in epoxy resin sample with large air gap under 8 and 20 kHz bipolar square-wave voltage. They exhibited the same characteristics as Figure 8, namely bush-like electrical trees, and the electrical trees tended to carbonize the surrounding region. Similarly, discharge damage in the air gap and a severely deformed air gap tip were also observed. However, the black spots appeared earlier with the increasing voltage frequency.

**Figure 10:**
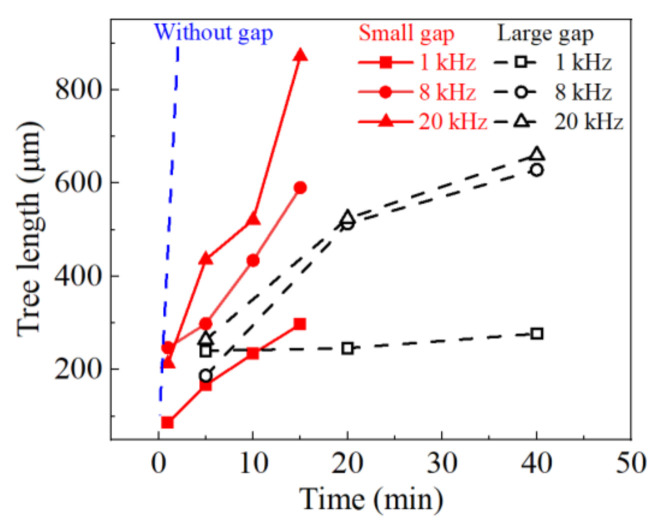
Electrical tree growing characteristics under bipolar square-wave voltage with air gap.

The length of the electrical tree can be defined as the distance from the farthest end of the electrical tree to the tip of the air gap, and the tree length with increasing time can be obtained, as shown in Figure 10. The slope of the line segment in the figure reflect the growth rate of electrical tree. It can be seen that, compared with the growth rate of electrical tree in the sample without air gap (as shown by the blue dotted line), the existence of the air gap significantly reduces the growth rate of electrical tree, and the larger the size of the air gap, the slower the growth rate. From the comparison of electrical tree growth under different voltage frequencies, in the range of 1~20 kHz, the increase in frequency will help the rapid growth of electrical tree, no matter the air gap is large or small.

### 3.3. Erosion Effect of Corona Discharge

**Figure 11:**
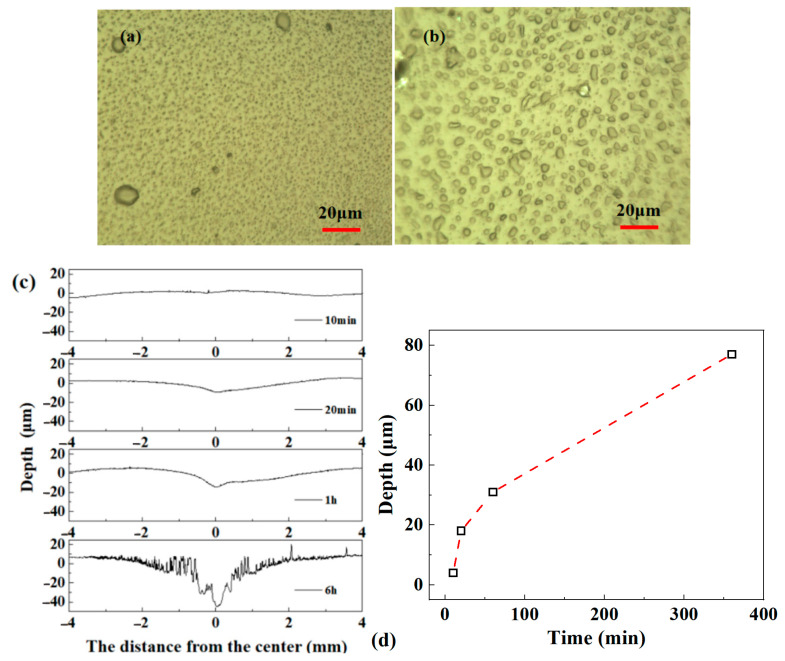
Surface morphology after erosion under bipolar square-wave voltage: (**a**) surface picture after 10 min erosion; (**b**) surface picture after 20 min erosion; (**c**) surface contour curve; (**d**) erosion rate at the center position.

In order to explore the corona damage characteristics in the air gap under high frequency bipolar square-wave voltage, the corona discharge experiments were conducted, and the voltage frequency was set at 3 kHz. For effectively judge the early deterioration characteristics, the test voltage cannot be too high in order to delay the surface damage speed. Before the test, the voltage was continuously increased, and it was found that the corona near the needle tip appeared above about 3.1~3.3 kV. Therefore, the test voltage amplitude was set at 4 kV. Figure 11 shows the surface morphology of epoxy resin sheet samples after discharge erosion.

The most obvious feature of discharge erosion on the sample surface is unevenness. The sample surface after corona for 10 min showed a large number of erosion spots with about 1 μm in size, as shown in Figure 11a. With the further extension of the corona time, the erosion spots increase in size, to about 4 μm, as shown in Figure 11b. For the overall understand on the corona damage, the surface contour curves of epoxy resin sample were obtained, and based on this, we can calculate the erosion rate under bipolar square-wave voltage at the center position of the samples, as shown in Figure 11d.

## 4. Discussion

**Figure 12:**
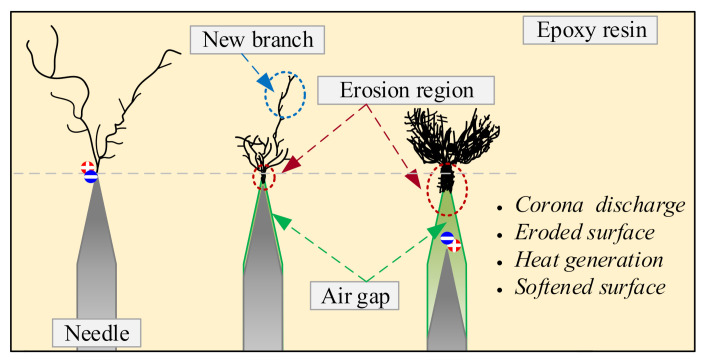
Schematic diagram of electrical treeing characteristics under high frequency bipolar square-wave voltage.

The growth of electrical tree in epoxy resin matrix should be a combination of molecular chain breakdown and air gap corona corrosion. Combined with the schematic diagram shown in Figure 12, the test results can be discussed from multiple perspective.

From the perspective of charge transport, under an AC electric field, the injection and extraction of charges are the key to the electrical treeing [21]. The energy generated by charge recombination and the local electric field distortion caused by polarity reversal are both causes of local breakdown [21]. Therefore, in this paper, the electrical tree in the sample without air gap was also considered to originate from the local breakdown of the epoxy insulation near the needle tip, and the electrical tree grows in the form of continuous molecular chain breakdown. The key difference from previous studies on the AC voltage electrical treeing is that the number of tree branches under high frequency bipolar square-wave voltage is very small, and the growth rate is very fast [7,13,22,23]. It can be explained from two aspects. One is that the number of voltage polarity reversals per unit time is very large, and thus, the charge recombination is frequent; hence, the continuous release of energy causes the scission of the molecular chains. Many test results showed that the breakdown strength of the insulation dielectrics decreased obviously with the increasing voltage frequency [24,25,26]. When the frequency increases from 5 kHz to 20 kHz, the breakdown voltage of epoxy resin drop to 56.5% of original values [24]. The second is that the sustaining time of the positive or negative voltage is very short under high frequency bipolar voltage. Ignoring the rising edge time, the sustaining times were approximately 500, 62.5 and 25 μs respectively under 1, 8 and 20 kHz voltage. Therefore, the injected charges can only accumulate on the surface layer and there is not enough time for the net charges to migrate inward. The electric field distortion in the local small region is very large and it is easy to induce breakdown. With the increasing frequency, the voltage stabilization time becomes shorter, and it is difficult to breakdown in multiple directions during a period time. Therefore, the electric tree has obvious directionality and the branch number of electrical tree becomes small.

From the perspective of the corona discharge in the air gap and the subsequent breakdown channel, it destroys the surrounding epoxy matrix. On the one hand, the diameters of the breakdown channels increase, which consequently increases the radius of curvature at the tip position. When the role of electric charge is not considered, the local electric field strength in the tip position can be approximately calculated by [25]:

(1) $$E=\frac{2V}{r\ln(1+4R/r)}$$

where *V* is the voltage, *r* is the pin radius and *R* is the pin-tip-plane separation.

Therefore, the increase of pin radius will weaken the electric field concentration. On the other hand, as the above-mentioned experiments have verified that the corona’s erosion on the surface of the epoxy material is uneven, that is, a lot of small-sized corrosion defects are generated. The increasing defects increases the possibility of electrical trees extending to the surroundings, which makes it easier to produce bush-type trees. In addition, the partial discharge under high-frequency AC voltage occurs within a short period of time after the voltage polarity is reversed; hence, higher voltage frequency means that the number of discharges increases proportionally [26,27,28]. Although the maximum partial discharge amplitude decreases with the increasing voltage frequency, the average partial discharge amplitude did not decrease when the voltage frequency is below 20 kH [26,29].

Long-term continuous discharge in the air gap will generate a lot of heat, and the higher voltage frequency will lead to a higher temperature rise [19,26]. Therefore, the heat accumulation is also one of the factors affecting the characteristics of electrical tree. This is a big difference between the two cases with and without air gap. Due to the poor thermal conductivity of epoxy [30], the heat cannot be effectively released, so the temperature in the air gap will continue to rise. Dynamic mechanical test results showed that the glass transition temperature of the epoxy sample used in this paper is about 100 °C [30]. Therefore, the epoxy matrix near the air gap will soften and deform, so that the sharp position becomes passivated, and the weakened local electric field slows the growth rate of the electric treeing. Due to the combined effect of multiple factors, the electrical tree under this type of voltage has its unique characteristics.

## 5. Conclusions

This paper studies the electrical tree in epoxy casting insulation under the high frequency bipolar square-wave voltage and focuses on the comparative study of the influence of the air gap on the electrical treeing. The effects of the voltage frequency on electrical treeing are also discussed. The main conclusions are as follows:

1. With the presence of air gap around the needle tip, the electrical tree in epoxy casting insulation under repetitive frequency bipolar square-wave voltage changes from branch-type to bush-type. At the same time, the growth rate of electrical tree significantly decreases, and the larger the air gap, the slower the growth rate.
2. In the range of 1~20 kHz, no matter whether with large air gap or small air gap, discharge in the air gap is more intense, and the growth rate of electrical tree branches increases with the increasing voltage frequency. Different degrees of erosion and deformation have appeared in the tip area of the air gap.
3. The multiple effects of discharge erosion, heat accumulation and charge transport determine the electrical tree characteristics. Among them, the spot-like erosion characteristics of the epoxy sample surface under the repetitive frequency bipolar square-wave voltage indicate that discharge will introduce a lot of defects and induce the possibility of multi-directional expansion of electrical trees.
